# Cardiovascular Outcomes Following Therapeutic Sclerostin Inhibition Compared With Alternative Anabolic Therapies: A Real‐World Propensity Score–Matched Analysis

**DOI:** 10.1155/joos/2813928

**Published:** 2026-07-30

**Authors:** Maxim John Levy Barnett, Justin Lam, Catherine Anastasopoulou

**Affiliations:** ^1^ Department of Endocrinology, Diabetes & Metabolism, Johns Hopkins Hospital, Baltimore, Maryland, USA, jhu.edu; ^2^ Department of Internal Medicine, Jefferson-Einstein Hospital, Philadelphia, Pennsylvania, USA; ^3^ Department of Endocrinology, Diabetes & Metabolism, Jefferson-Einstein Hospital, Philadelphia, Pennsylvania, USA

**Keywords:** cardiovascular safety, evenity, MACE, osteoporosis, romosozumab, sclerostin

## Abstract

**Introduction:**

Romosozumab is a dual antiresorptive/anabolic monoclonal antibody against sclerostin, approved for osteoporosis. Largely driven by the ARCH trial, unexpected concerns for cardiovascular safety arose, mandating a black‐box warning. Despite numerous subsequent investigations, the true nature of this association is unelucidated.

**Materials and Methods:**

Real‐world data were analyzed through TriNetX network, further clarifying this association. Over 1 year, we analyzed patients aged > 50 with osteoporosis, exposed to romosozumab (Cohort A) or teriparatide/abaloparatide (Cohort B), with propensity score matching. We did not exclude patients with outcomes of interest prior to the index event.

**Results:**

We observed a significant association with lower hazard ratios among four‐ (HR 0.441, 95% CI 0.0.376–0.638, *p* < 0.0001) and three‐point major adverse cardiovascular events (HR 0.624, 95% CI 0.567–0.688, *p* < 0.0001), with improved survival probabilities among Cohort A. Romosozumab participants continued to demonstrate a significantly lower hazard (and improved survival probabilities) of myocardial infarction, heart failure, and death, but no significant difference was observed with respect to cerebrovascular accidents. Subsequent E‐value sensitivity analyses suggested moderate robustness to unmeasured confounding. Further subgroup analyses were performed over two and five years of follow‐up, among patients aged 50–64 and > 65, along with male‐ and female‐only cohorts.

**Conclusion:**

The true association between romosozumab and cardiovascular events remains unknown. Additional studies of a prospective nature are required to investigate this further. These findings do not demonstrate a clear increased cardiovascular risk signal in a real‐world setting; however, they should be interpreted as associative rather than causal and are not sufficient to change current regulatory recommendations.

## 1. Introduction

Truswell et al. described an unusual phenotype among a South African cohort of Afrikaner descent in 1958. In his seminal paper, Truswell described features of hyperostosis, osteosclerosis, facial distortion (with cranial nerve entrapment), syndactyly, and tall stature; collectively, these findings were termed *sclerosteosis* [1]. In 1955, van Buchem et al. described a milder phenotype among a Dutch family, demonstrating bone overgrowth and cranial nerve compression but without additional features, along with a benign clinical course. Although initially referred to as *hyperostosis corticalis generalisata familiaris*, it is better known as *van Buchem disease* [1]. Despite the pronounced skeletal thickening, however, affected individuals demonstrated a markedly reduced risk for fractures [1]. It was not until around half a century later that the gene responsible for these diseases (*SOST* gene, Chromosome 17) and its protein (sclerostin) were discovered [2].

**FIGURE 1:**
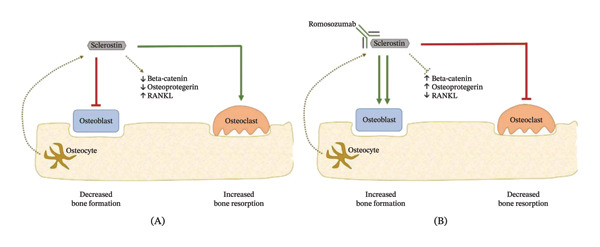
Permission obtained from [4]. Under normal circumstances, sclerostin is released by osteocytes, binding to low‐density lipoprotein receptor‐related proteins 5 and 6 (LRP5 and 6), alongside various coreceptors; this interaction prevents the interaction of wingless/integrated (wnt) ligands with osteoblast receptors. Such a blockade allows for downstream activation of the “destruction activity complex,” promoting ubiquitination and degradation of beta‐catenin, alongside the expression of receptor activator of nuclear factor kappa‐beta ligand (RANKL), with subsequent inhibition of osteoprotegerin. In the absence of sclerostin, however, wnt ligands interact with osteoblastic receptors (LRP 5 and 6, as well as Frizzled coreceptors), preventing the “destruction activity complex,” which promotes intracellular accumulation and nuclear translocation of beta‐catenin, with resulting activation of transcription factors responsible for osteoblast proliferation (alongside osteoprotegerin production). Consequently, however, the effect of sclerostin inhibition is two‐fold—enhanced bone formation (anabolic activity) and reduced bone resorption (antiresorptive activity). Abbreviation: RANKL = receptor‐activator of nuclear factor kappa‐beta ligand.

Sclerostin, a 22 kDa (190‐amino acid glycoprotein), is the primary target of the novel osteoporotic agent romosozumab (Figure 1) [3, 4]. Romosozumab‐aqqg (trade name Evenity) is a humanized IgG_2_ monoclonal antibody, developed by Amgen and UCB Pharma, approved for the treatment of severe osteoporosis in patients at high risk for fractures [5]. Clinical trials have demonstrated romosozumab to significantly increase bone mineral density and reduce the incidences of vertebral, hip, and nonvertebral fractures [6]. The standard regimen consists of 210 mg administered subcutaneously once monthly, delivered via two consecutive injections of 105 mg each [6]. The approximate cost of romosozumab per Medicare beneficiary for 12 months is around $5500 [7]. Between 2019 and 2020, there were an estimated 15,000 prescriptions in the United States [8]. At present, treatment duration is limited to 12 months, as the anabolic effects diminish thereafter; consequently, patients must transition to antiresorptive therapy to maintain their bone mineral density.

The Japanese Pharmaceuticals and Medical Devices Agency (PMDA) was the first to approve romosozumab for the treatment of postmenopausal osteoporosis in March 2019 [9]. Subsequently, the U.S. Food and Drug Administration (FDA) granted approval in April 2019, followed by European Medicines Agency (EMA) in December 2019 [9]. While most regulatory approvals worldwide have focused on postmenopausal women at high risk for fracture, Japan, alongside South Korea and Australia, remains among the only countries to have extended the indication to include men with osteoporosis [10]. The approval of romosozumab by the FDA was based on the results of two randomized controlled trials, FRAME (Fracture Study in Postmenopausal Women with Osteoporosis) and ARCH (Active‐Controlled Fracture Study in Postmenopausal Women with Osteoporosis at High Risk) [11]. Although potent, its clinical adoption has been tempered by concerns regarding cardiovascular safety. In 2016, the FDA and EMA withheld marketing authorization following preliminary data from the ARCH trial demonstrating an imbalance in serious cardiovascular events [12]. After subsequent safety reviews, these agencies ultimately granted approval in 2019, with a mandated boxed warning [12]. Notably, in Japan, romosozumab did not initially include a warning; however, the product was subsequently revised by the PMDA to incorporate comparable warnings regarding cardiovascular events [12].

Uncertainty persists regarding the mechanism and magnitude of cardiovascular risks associated with romosozumab. Subsequent randomized controlled trials, meta‐analyses, and post hoc evaluations continue to provide inconsistent conclusions. Given the unresolved questions and limitations of trial‐based evidence in reflecting real‐world populations, further postmarketing surveillance and observational studies are critical. We perform a propensity score–matched retrospective cohort analysis to evaluate the association, or lack thereof, between romosozumab exposure and cardiovascular events in a real‐world setting. This study aims to clarify whether the cardiovascular safety concerns observed in controlled trials persist outside the confines of a randomized study population, thereby informing future clinical decision‐making and regulatory considerations.

## 2. Materials and Methods

### 2.1. Study Design and Data Source

The study was reported in accordance with the STROBE checklist (Supporting Appendix 1). We utilized the TriNetX Global Collaborative Network, to retrospectively analyze de‐identified electronic healthcare records from more than 160 healthcare organizations (with over 200 million patients), among inpatient and outpatient settings. Currently, TriNetX is available in 21 countries; however, most of the data reported are from the United States. Using deidentified and anonymized data, informed consent was not required for this study, which was exempt from an institutional review board approval. We utilized the International Statistical Classification of Diseases and Related Health Problems, 10th Revision (ICD‐10), alongside the United States National Library of Medicine (RxNorm) codes.

### 2.2. Study Population

We defined two cohorts for analysis; Cohort A was defined based on the presence of osteoporosis, age above 50 years, and treatment with romosozumab; conversely, cohort B was defined as the presence of osteoporosis in patients above the age of 50 who were exposed to either teriparatide or abaloparatide (and without exposure to romosozumab) (Table 1). All genders were included, as well as inpatient and outpatient data, without a geographic restriction. As romosozumab was not available until 2019, this analysis was performed from January 2019 until present (October 2025). To approximate treatment adherence, patients included in Cohort A were required to have at least five administrations of romosozumab. Due to the black‐box warning, romosozumab patients are often preselected for their low cardiovascular risk, creating a relative “healthy user bias”; as such, we did not exclude patients with prior cardiovascular disease. Our comparator group consisted of patients exposed to teriparatide or abaloparatide; unlike antiresorptive therapies, these agents are often used in patients with comparable disease severity, allowing for a more appropriate comparison of outcomes in patients eligible for advanced osteoporosis treatment. Moreover, teriparatide and abaloparatide are relatively neutral with respect to cardiovascular safety.

**TABLE 1:** ICD‐10 and RxNorm inclusion and exclusion criteria.

Cohort A	Cohort B
ICD‐10 inclusion criteria: age ≥ 50 + M80 (osteoporosis with current pathological fracture); M80.0 (age‐related osteoporosis with current pathological fracture); M80.00 (age‐related osteoporosis with current pathological fracture, unspecified site); M80.00XA (age‐related osteoporosis with current pathological fracture, unspecified site, initial encounter for fracture); M80.00XD (age‐related osteoporosis with current pathological fracture, unspecified site, subsequent encounter for fracture with routine healing); M80.05 (age‐related osteoporosis with current pathological fracture, femur); M80.08 (age‐related osteoporosis with current pathological fracture, vertebra(e)); M80.08XA (age‐related osteoporosis with current pathological fracture, vertebra(e), initial encounter for fracture); M81 (osteoporosis without current pathological fracture); M81.0 (age‐related osteoporosis without current pathological fracture); Z87.310 (personal history of [healed] osteoporosis fracture)	ICD‐10 inclusion criteria: age ≥ 50 + M80 (osteoporosis with current pathological fracture); M80.0 (age‐related osteoporosis with current pathological fracture); M80.00 (age‐related osteoporosis with current pathological fracture, unspecified site); M80.00XA (age‐related osteoporosis with current pathological fracture, unspecified site, initial encounter for fracture); M80.00XD (age‐related osteoporosis with current pathological fracture, unspecified site, subsequent encounter for fracture with routine healing); M80.05 (age‐related osteoporosis with current pathological fracture, femur); M80.08 (age‐related osteoporosis with current pathological fracture, vertebra(e)); M80.08XA (age‐related osteoporosis with current pathological fracture, vertebra(e), initial encounter for fracture); M81 (osteoporosis without current pathological fracture); M81.0 (age‐related osteoporosis without current pathological fracture); Z87.310 (personal history of [healed] osteoporosis fracture)

RxNorm inclusion criteria: romosozumab (2123126)	RxNorm inclusion criteria: Teriparatide (32915) or abaloparatide (1921069)
	RxNorm exclusion criteria: Romosozumab (2123126)

### 2.3. Statistical Analysis

The index event was defined as the first date a patient fulfilled criteria for cohort entry. We employed one‐to‐one propensity score matching for *n* = 19 variables to control for confounding (age at index, gender, ethnicity, race, tobacco use [Z72.0], essential hypertension [I10], chronic kidney disease [N18], diseases of the circulatory system [I00‐I99], family history of ischemic heart disease and other diseases of the circulatory system [Z82.49], family history of stroke [z82.3], serum low‐density lipoprotein (LDL) cholesterol, serum hemoglobin A1c, and cardiovascular medications), with the greedy/nearest neighbor method with 0.1 calipers of pooled standard deviations; furthermore, TriNetX software incorporates logistic regression for propensity score matching. Baseline characteristics were obtained among both cohorts, with a two‐sided *t*‐test utilized to compare for baseline differences; statistical significance was defined as a two‐sided *p* value < 0.05. Covariate balance after propensity score matching was evaluated using standardized mean differences (SMDs), with values < 0.1 indicative of acceptable balance.

Laboratory variables demonstrated incomplete availability, reflecting expected missingness in electronically derived data. Analyses were conducted using available‐case data within the TriNetX platform. No formal imputation of missing values was performed. Propensity score matching was therefore based on observed covariates, with patients retained in the analysis despite incomplete data for certain variables. As a result, matching reflects available clinical information rather than complete covariate profiles.

Time‐to‐event outcomes were analyzed with a Kaplan–Meier survival analysis, using both the log‐rank test and Cox proportional hazards modeling. The log‐rank test (reported as *χ* ^2^ and degrees of freedom [df]) was used to compare overall survival distributions between Cohorts A and B across the study period; Cox proportional hazards models were applied for the estimation of the hazard ratio (HR) and 95% confidence intervals (CI), providing both magnitude and direction of effect (under proportional hazards assumption). Both metrics are presented, as discrepancies between log‐rank and Cox *p* values can occasionally arise from model assumptions and small event counts with subgroup analyses, reporting both ensured transparency by displaying the statistical significance of both the survival curve and corresponding effect size. A two‐sided *p* value < 0.05 was considered statistically significant (alongside a critical *χ* ^2^ of 3.841 when df = 1). For sensitivity analyses, an E‐value was calculated (≥ 2.0 moderately robust and ≥ 3.0 strongly robust).

### 2.4. Outcome

Outcome definitions were based on ICD‐10 diagnostic codes selected to represent clinically significant cardiovascular events. These codes were chosen to prioritize specificity and consistency with commonly used definitions in large administrative and electronic health record–based studies. While broader code groupings could capture additional events, the selected codes were intended to reflect clearly defined, clinically relevant outcomes. The primary objectives were both four‐point major adverse cardiovascular events (MACE) (myocardial infarction [ICD‐10: I21], heart failure [ICD‐10: I50.21], cerebrovascular accident [ICD‐10: I63.50], and death) and three‐point MACE (myocardial infarction [ICD‐10: I21], heart failure [ICD‐10: I50.21], and cerebrovascular accident [ICD‐10: I63.50]). Secondary outcomes included individual MACE components (myocardial infarction, heart failure, cerebrovascular accident, and death). Outcomes were assessed during follow‐up from one day postindex event over 1 year.

## 3. Results

Before matching, we identified *n* = 53,638 (Cohort A: *n* = 13,400; Cohort B: *n* = 40,238). Following propensity score matching, the final analytic cohort included *n* = 26,800 patients (*n* = 13,400 per group). Baseline characteristics were well balanced between cohorts after matching. The groups were similar in age at index event (71.1 ± 8.55 vs. 71.2 ± 8.66 years), sex distribution (female: 97.43% vs. 97.40%), and race, including White participants (75.78% vs. 76.04%) and African American participants (1.37% vs. 1.06%). Use of cardiovascular medications (78.55% vs. 78.81%), prevalence of cardiovascular disease (67.19% vs. 66.95%), essential hypertension (47.34% vs. 47.51%), and LDL cholesterol levels (102 ± 34.1 vs. 100 ± 35 mg/dL; SMD < 0.1) were also comparable between groups. Cohort A demonstrated slightly higher proportions of chronic kidney disease (11.66% vs. 10.88%; SMD < 0.1), family history of cardiovascular disease (9.73% vs. 8.93%; SMD < 0.1), tobacco use (3.49% vs. 2.96%; SMD < 0.1), and family history of stroke (2.19% vs. 1.72%; SMD < 0.1); however, all corresponding SMDs were < 0.1, indicating that these differences were negligible. Covariate balance was assessed using SMDs, with values < 0.1 indicating adequate balance. Postmatching, nearly all covariates demonstrated excellent balance (SMD < 0.1), supporting the adequacy of the matching procedure. A small residual imbalance was observed for hemoglobin A1c (5.71% ± 0.78 vs. 5.79% ± 0.91; SMD = 0.1017), which marginally exceeded the conventional threshold (Supporting Appendix 2). Notably, several variables demonstrated statistically significant *p* values despite having SMDs < 0.1, reflecting the influence of large sample size and indicating negligible differences rather than meaningful imbalance. Taken together, these findings indicate that residual imbalances were minimal and unlikely to meaningfully impact the validity of the matched comparison.

### 3.1. Primary Outcomes

**FIGURE 2:**
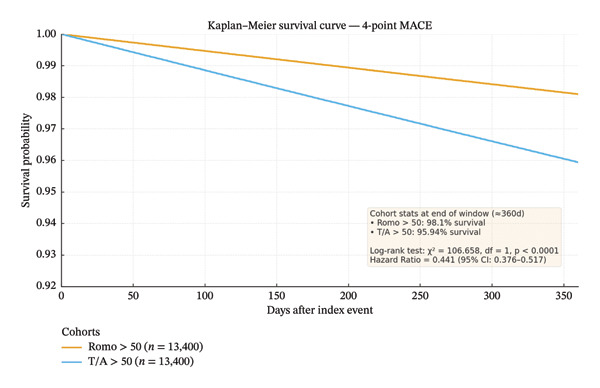
Kaplan–Meier survival curve (four‐point major adverse cardiovascular events).

With four‐point MACE, the Kaplan–Meier survival analysis demonstrated a statistically significantly greater event‐free survival in Cohort A; over 1 year, *n* = 222 events occurred among Cohort A, compared to *n* = 466 in Cohort B. The estimated survival probability at the end of the observation window was 98.1% versus 95,937%, respectively (absolute risk difference ∼ 2.2%). A log‐rank test confirmed a significant difference between survival curves (*χ* ^2^ = 106.658, df = 1, *p* < 0.0001). The HR for the outcome was 0.441 (95% CI 0.376–0.517, *p* < 0.0001) (Figure 2). The proportional hazards assumption was tested and satisfied.

**FIGURE 3:**
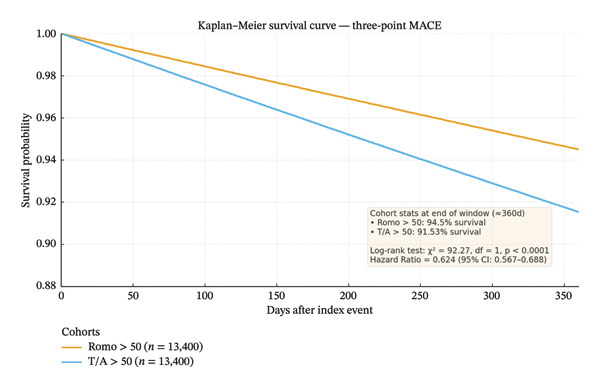
Kaplan–Meier survival curve (three‐point major adverse cardiovascular events).

Similarly, with three‐point MACE, the Kaplan–Meier survival analysis demonstrated a significantly greater event‐free survival in Cohort A. Over the study period, *n* = 686 events occurred in Cohort A, compared to *n* = 1008 events in Cohort B. The estimated survival probability at the end of the observation window was 94.5% versus 91.528% (absolute risk difference ∼ 3.0%). A log‐rank test confirmed a significant difference between survival curves (*χ* ^2^ = 92.27, df = 1, *p* < 0.0001) (Figure 3). The HR for the outcome was 0.624 (95% CI 0.567–0.688, *p* < 0.0001). The proportional hazards assumption was tested and satisfied. For both four‐ and three‐point MACE, despite statistically significant HRs, the absolute risk differences were modest, reflecting the relatively low overall event rates.

### 3.2. Secondary Outcomes

For heart failure, *n* = 618 events occurred in Cohort A versus *n* = 887 in Cohort B, with an event‐free survival of 95.057% versus 92.545%. The difference was significant (log‐rank test: *χ* ^2^ = 73.223, df = 1, *p* < 0.0001) with a HR of 0.641 (95% CI 0.578–0.710, *p* = 0.0002). For all‐cause mortality, *n* = 104 events occurred in Cohort A compared to *n* = 246 in Cohort B, with a significant difference in survival probabilities of 99.078% and 97.803% (log‐rank test: *χ* ^2^ = 68.265, df = 1, *p* < 0.0001) and a HR of 0.394 (95% CI 0.313–0.495, *p* < 0.0001). With acute myocardial infarction, *n* = 103 versus *n* = 197 events occurred, with a significant difference in survival (99.14% versus 98.307%) (log‐rank test: *χ* ^2^ = 37.094, df = 1, *p* < 0.0001). Moreover, the HR was 0.484 (95% CI 0.382–0.615, *p* = 0.0025). For the final individual outcome of cerebrovascular accident, *n* = 16 versus *n* = 31 events occurred, with survival probabilities of 99.867% versus 99.741%. The log‐rank test demonstrated a statistically significant difference (*χ* ^2^ = 5.801, df = 1, *p* = 0.0160). However, the corresponding Cox proportional hazards model did not reach statistical significance (HR 0.484, 95% CI 0.265–0.885, *p* = 0.1007), indicating inconsistency between statistical approaches. This discrepancy likely reflects the low number of events and instability of estimates in sparse data. Proportional hazards assumptions were met across all analyses (Table 2).

**TABLE 2:** Primary and secondary outcomes.

Outcome	*χ* ^2^	df	Log‐rank *p* value	HR (95% CI)	HR *p* value	Interpretation
Four‐point MACE (primary outcome)	106.658	1	< 0.0001	0.441 (0.376–0.517)	< 0.0001	Significant
Three‐point MACE (primary outcome)	92.27	1	< 0.0001	0.624 (0.567–0.688)	< 0.0001	Significant
Acute heart failure (secondary outcome)	73.223	1	< 0.0001	0.641 (0.578–0.710)	0.0002	Significant
All‐cause mortality (secondary outcome)	68.265	1	< 0.0001	0.394 (0.313–0.495)	< 0.0001	Significant
Acute myocardial infarction (secondary outcome)	37.094	1	< 0.0001	0.484 (0.382–0.615)	0.0025	Significant
Cerebrovascular accident (secondary outcome)	5.801	1	0.0160	0.484 (0.265–0.885)	0.1007	Log‐rank significant, HR not significant

*Note:* χ^2^ = chi‐squared.

Abbreviations: CI = confidence interval, HR = hazard ratio, MACE = major adverse cardiovascular event.

### 3.3. Sensitivity Analysis

Among the primary and secondary outcomes, we employed an E‐value sensitivity analysis to assess the robustness of the HRs (quantifying the minimum strength of an association required by an unmeasured confounder [need to have with both exposure and outcome], beyond the measured covariates, to fully explain away the observed association). This included an E‐value point estimate (minimum association required to reduce the observed association to the null) and an E‐value CI limit (association required to move the CI to include the null). The E‐value point estimated ranged from 2.07 to 3.92, with CI limits between 1.67 and 3.25. The highest robustness was observed with four‐point MACE (*E* = 3.92; limit = 3.25) and death (*E* = 3.68; limit = 3.01), which were least likely to be driven by unmeasured bias. Outcomes such as heart failure and three‐point MACE demonstrated moderate robustness (E‐values 2.0–2.5) with cerebrovascular accidents showing limited robustness (*E* = 2.07, limit = 1.67), consistent with the nonsignificant HR (Table 3).

**TABLE 3:** E‐Value sensitivity analysis.

Outcome	E‐value point estimate	E‐value confidence interval limit	Robustness
Four‐point MACE (primary outcome)	3.96	3.28	Strong
Three‐point MACE (primary outcome)	2.59	2.27	Moderate
Acute heart failure (secondary outcome)	2.49	2.17	Moderate
All‐cause mortality (secondary outcome)	4.51	3.46	Strong
Acute myocardial infarction (secondary outcome)	3.55	2.63	Strong
Cerebrovascular accident (secondary outcome)	3.55	1.51	Strong

Abbreviation: MACE = major adverse cardiovascular event.

### 3.4. Subgroup Analysis

All endpoints (apart from cerebrovascular accidents) remained statistically significant and directionally unchanged over 2 and 5 years of follow‐up (Supporting Appendix 3). Male subgroup analysis was underpowered for significant results (Supporting Appendix 4); however, with the female‐only subgroup, all outcomes were significantly reduced (including cerebrovascular accidents) (Supporting Appendix 5). Analysis of ages 50–64 led to a significant reduction in four‐ and three‐point MACE, as well as heart failure (with death, myocardial infarction, and cerebrovascular accidents too low for analyses) (Supporting Appendix 6); conversely, above age 65, there were significant reductions in four‐ and three‐point MACE, along with heart failure, myocardial infarction, and death (cerebrovascular accidents were not statistically significant) (Supporting Appendix 7).

## 4. Discussion

In this large real‐world cohort study, following propensity score matching and 1 year of follow‐up, our findings suggest that romosozumab exposure is associated with lower observed rates of MACEs compared with alternative anabolic therapies. This was observed across composite and individual MACE outcomes (confirmed by log‐rank and Cox proportional hazards modeling). The associations were most robust for four‐ and three‐point MACE, death, and heart failure, with statistical significance (but comparatively modest) protection from acute myocardial infarction. The modest absolute risk differences observed suggest that the clinical impact may be smaller than implied by relative estimates alone. E‐value analyses suggest moderate robustness to unmeasured confounding; however, these values do not exclude the possibility that residual confounding could account for the observed associations. Accordingly, the results should be interpreted with caution.

The findings for cerebrovascular events should be interpreted with substantial caution. The low number of observed events markedly limits statistical power and results in imprecise and potentially unstable effect estimates (increasing susceptibility to both Type I and Type II errors). This is reflected in the inconsistency between log‐rank and Cox proportional hazards analyses and suggests that the observed associations may be unreliable. Accordingly, these results should not be considered definitive, likely reflecting random variation rather than a true underlying effect. Although subgroup analyses in female participants demonstrated a statistically significant reduction, this contrasts with the primary analysis and may reflect statistical variability, multiple comparisons, or potential effect modification by sex. While sex‐specific differences in thrombotic risk could be hypothesized, there is insufficient evidence to support a definitive mechanistic explanation, and the apparent divergence may reflect either biological heterogeneity or inadequate statistical power. Given that cerebrovascular events are included in regulatory safety warnings alongside myocardial infarction, these findings underscore the need for cautious interpretation. Our results do not provide sufficient evidence to refute current safety concerns related to stroke risk, and further investigation in adequately powered studies with adjudicated endpoints is required.

Our findings differ from several randomized trials and meta‐analyses, which have generally demonstrated neutral or potentially increased cardiovascular risk. This discrepancy may reflect differences in study design, population characteristics, and real‐world prescribing patterns. Notably, the magnitude of the observed associations—particularly HRs substantially below unity for several outcomes—may exceed what would be considered biologically plausible for a pharmacologic intervention of this nature. As such, these findings should be interpreted cautiously, as they may in part reflect residual confounding, selection bias, and other systematic sources of error rather than a true cardioprotective effect.

There are two similar studies reported in the medical literature analyzing cardiovascular outcomes with the direct comparison of romosozumab to anabolic agents (teriparatide/abaloparatide); while both studies excluded patients with a history of cardiovascular events prior to the index event, our study differs with the inclusion of such patients to attempt to overcome the “healthy‐user bias.” Furthermore, we differ in analyzing outcomes over one, two, and 5 years (to capture delayed cardiovascular events). Masuda et al. among a Japanese population compare romosozumab to teriparatide, failing to identify a statistically significant difference in MACE risk [13]. Stokar and Szalat furthermore compare romosozumab to teriparatide/abaloparatide, noting statistically significant decreases in three‐point MACE and its individual components of myocardial ischemia, cerebrovascular events, and death [14]. The discrepancy between our findings and those of Stokar and Szalat with respect to cerebrovascular events likely reflects methodological and statistical differences [14].

### 4.1. Initial Cardiovascular Safety Concerns

The ARCH trial compared romosozumab (followed by alendronate) to alendronate monotherapy in *n* = 4093 postmenopausal women with severe osteoporosis and noted an unexpected imbalance in cardiovascular events during the first year of treatment (*n* = 50 [2.5%] versus *n* = 39 [1.9%]), although this was not statistically significant [15]. Further subgroup analyses solely noted a significant increase in cardiac ischemia (*n* = 16 [0.8%] versus *n* = 6 [0.3%]) (OR 2.54; 95% CI 1.03–6.77), alongside a numerically greater (but statistically insignificant) incidence of cerebrovascular events [15]. One year prior to ARCH, however, the FRAME trial (with a larger cohort of *n* = 7180 postmenopausal women) compared romosozumab to placebo (with open‐label denosumab in both groups) [16]. In FRAME, reassuringly, no cardiovascular safety signal was demonstrated; at 12 and 24 months, cardiovascular events remained similar between cohorts, along with the absence of differences in MACE, myocardial infarction, or cerebrovascular accidents [16]. Two subsequent, smaller, randomized controlled trials have provided conflicting results. The STRUCTURE trial (romosozumab versus teriparatide in postmenopausal women with osteoporosis transitioning from oral bisphosphonate therapy) analyzed *n* = 436 postmenopausal women, comparing romosozumab to teriparatide; low event rates are noted, with no significant difference in cardiovascular outcomes [17]. The BRIDGE trial (a Phase III Randomized Placebo‐Controlled Trial to Evaluate Efficacy and Safety of Romosozumab in Men with Osteoporosis), however, assessed *n* = 245 men with osteoporosis (comparing romosozumab to placebo), noting statistically insignificant increases in event rates with romosozumab (Table 4) [18].

**TABLE 4:** Comparison of the ARCH, FRAME, BRIDGE, and STRUCTURE trials.

Number of participants	ARCH	FRAME	BRIDGE	STRUCTURE
	*n* = 4093	*n* = 7180	*n* = 245	*n* = 436
Gender	Female	Female	Male	Female
Event group	Romosozumab for 1 year (followed by transition to alendronate)	Romosozumab (open‐label transition to denosumab after 1 year)	Romosozumab	Romosozumab (previously on alendronate for at least 3 years)
Control group	Alendronate	Placebo (open‐label transition to denosumab after 1 year)	Placebo	Teriparatide (previously on alendronate for at least 3 years)
Cardiac outcomes	During the first year of treatment, romosozumab group experienced *n* = 50 (2.5%) cardiovascular events compared to alendronate *n* = 39 (1.9%), although not statistically significant (OR 1.31; 95% CI 0.85–2.00). After subgroup analyses, excess risk driven by cardiac ischemia (*n* = 16 [0.8%] versus *n* = 6 [0.3%]) (OR 2.54; 95% CI 1.03–6.77), along with a statistically insignificant increase in cerebrovascular accidents. Statistically insignificant lower rates of peripheral vascular disease, noncoronary revascularization, and heart failure with romosozumab.	At 12 months of treatment, cardiovascular events remained similar between romosozumab (1.2%) and placebo (1.1%) (HR 1.0; 95% CI 0.66–1.50), as well as at 24 months after transitioning to denosumab (2.3% versus 2.2%) without an observed difference in MACE (0.8% versus 0.8%; HR 1.03, 95% CI 0.62–1.72), myocardial infarction (0.3% versus 0.2%), or cerebrovascular accidents (0.2% versus 0.3%).	Statistically insignificant numerical increase in adjudicated serious cardiovascular events in romosozumab (*n* = 8 [4.9%]) compared to placebo (*n* = 2 [2.5%]). Events included cardiac ischemia (*n* = 3 [1.8%] versus *n* = 0), cerebrovascular accidents (*n* = 3 [1.8%] versus *n* = 1 [1.2%]), and heart failure (*n* = 1 [0.6%] versus *n* = 0).	Events not reported apart from nonstatistical difference in cardiac outcomes.

### 4.2. Interpretation of Trials

At first glance, there appears to be a concern for imbalance of cardiovascular events among romosozumab treatment; however, these results must be cautiously analyzed. First and foremost, these trials were not designed to investigate cardiovascular events (but rather bone mineral density and fracture risk); as such, these trials were underpowered (with a low number of participants and events, alongside wide CIs) [19]. As the primary outcome was not that of cardiovascular events, meticulous baseline data (such as a fasting lipid panel, C‐reactive protein, electrocardiography, among other cardiovascular risk markers) were not collected, limiting the ability of inferring causality [20]. As an aside, the FDA requires at least 25% of participants to be enrolled from the United States (for drug approval); notably, however, the ARCH and FRAME trials included 2.5% of patients from the United States and Canada (with similar populations such as Western Europe and Australia representing a further 14%) [21]. Such differences in access to (and delivery of) healthcare, education, activity, and diet across the various populations must be considered.

Despite a statistically significant increase in cardiovascular events within the ARCH trial, it is rather reassuring that the larger FRAME trial did not demonstrate any cardiovascular signal. A peculiar pattern of cardiovascular events is noted within the ARCH trial; during the first 3 months, there are no cardiovascular events among the alendronate cohort, followed by fewer cardiovascular events for the remainder of the 12 months [20]. When participants transitioned to alendronate after romosozumab, however, one would expect a decline in events; however, the event rates remained consistent without an abrupt change [20]. Moreover, as vascular calcification is not an acute process, the separation of alendronate from romosozumab within 3 months is more likely to be due to chance [20].

Within the medical literature, there are inconsistencies with respect to a cardioprotective role of bisphosphonates. It is therefore plausible that the MACE events noted in the ARCH trial were imbalanced due to a reduction in cardiovascular risk from alendronate (rather than as a direct result of romosozumab) and could furthermore explain why the imbalance was limited to ARCH (and not FRAME) [20]. This theory, however, does not appear plausible, with numerous meta‐analyses subsequently failing to demonstrate a cardioprotective role of bisphosphonates, as well as no biologically plausible explanation (theories include action upon mevalonate pathway and uptake by calcified plaque to promoting endothelial the function and stabilize active plaque) [22]. Within the ARCH trial, participants who were switched to alendronate after completion of romosozumab did not demonstrate a reduction in cardiovascular events. Reid further demonstrates that the MACE incidence in the alendronate group of the ARCH trial was identical to both groups within the FRAME trial [23].

The different populations included among each trial and study designs limit the ability to compare to one another. The mean ages in the ARCH, FRAME, and BRIDGE trials were 74, 71, and 72, respectively, suggesting that ARCH participants were older and were more likely to have cardiovascular disease and be susceptible to events [23]. While the ARCH trial included patients with severe osteoporosis, the FRAME trial included patients with a range of osteoporosis (as evidenced by more than 95% of ARCH participants having a prevalent vertebral fracture, compared to 18% in the FRAME trial), which could suggest that ARCH trial participants were less healthy [24]. Among the ARCH trial, 73% of participants had previously documented cardiovascular disease, compared to 65% (FRAME) and 66% (BRIDGE) [21]. Comparing ARCH to FRAME, hypertension (60% versus 53%), prior cerebrovascular disease (8% versus 5%), ischemic heart disease (13.5% versus 9%), heart failure (4% versus 2.5%), and atrial fibrillation (4% versus 2%) were all more prevalent among ARCH participants [14]. Moreover, ARCH participants were more likely to be prescribed cardiovascular (62% versus 57%) and antithrombotic medications (28% versus 23%) compared to FRAME participants [19]. Among ARCH participants that experienced cardiovascular events, nearly 90% had a history of either cardiovascular disease or at least one risk factor; moreover, there appeared to be an imbalance between ARCH cohorts, with more participants in the romosozumab arm above 75 years of age (66% versus 57.9%), former or current smokers (40% versus 31.6%), and those with hypercholesterolemia (50% versus 36.8%) [21]. A similar finding is noted in the BRIDGE trial, with most participants who experienced a cardiovascular event having preexistent cardiovascular disease (77.3% in the romosozumab cohort compared to 71.6% in the placebo group); counterintuitively, romosozumab participants appeared less likely to receive cardioprotective agents (57.1% versus 61.7%) [14]. With the discordant findings among these trials, numerous meta‐analyses have been performed, each yielding varying results (Table 5) [25–35].

**TABLE 5:** Meta‐analyses investigating romosozumab and cardiovascular outcomes.

Author	Year	Outcome
FDA [25]	2018	• ARCH, FRAME, and BRIDGE:
		○ Positively adjudicated cardiovascular severe adverse events for 12 months (double‐blind): HR 1.17 (95% 0.88–1.56)
		○ Overall study periods positively adjudicated cardiovascular severe advents: HR 1.06 (95% 0.89–1.25)
		○ Pooled MACE over first 12 months: HR 1.14 (95% CI 0.94–1.39)

Kaveh et al. [26]	2020	• Meta‐analysis of FRAME and BRIDGE:
		○ Adjudicated cardiovascular serious events: OR 1.12 (95% CI 0.75–1.69)
		○ Adjudicated cardiovascular death: OR 1 12 (95% CI 0.57–2.17)

Bovjin et al. [27]	2020	• Meta‐analysis of the ARCH and BRIDGE:
		○ MACE: OR: 2.98 (95% CI 1.18–7.55)
		• Meta‐analysis of ARCH, BRIDGE, and FRAME:
		○ MACE: OR: 1.54 (95% CI 0.90–2.64)

Lv et al. [28]	2020	• Romosozumab compared to active treatment or placebo:
		○ Composite cardiovascular outcome: OR 1.26 (95% 0.95–1.68)
		▪ Myocardial infarction: RR 1.39 (95% CI 0.72–2.69)
		▪ Stroke: RR 1.46 (95% CI 0.86–2.49)
		▪ Cardiovascular death: RR 1.36 (95% CI 0.67–2.74)
		▪ Heart failure: RR 1.26 (95% CI 0.66–2.42)
		▪ Atrial fibrillation: RR 1.12 (95% CI 0.49–2.54)
		○ Three‐point MACE: OR 1.41 (95% CI 0.99–2.02)
		○ Four‐point MACE: OR 1.39 (95% CI 1.01–1.90). (Sensitivity analysis led to nonsignificant four‐point MACE [OR 1.36, 95% CI 0.99–1.87])

Haändel et al. [29]	2023	• Romosozumab versus placebo:
		○ All‐cause mortality: RR 0.81 (95% CI 0.22–2.96)
		• Romosozumab versus bisphosphonates:
		○ All‐cause mortality: RR 0.98 (95% CI 0.74–1.31)
		• Romosozumab versus teriparatide
		○ All‐cause mortality: RR 0.82 (95% CI 0.10–6.62)

Choi et al. [30]	2023	• Romosozumab versus placebo:
		○ Composite cardiovascular adverse events: OR 1.16 (95% CI 0.82–1.65)
		○ MACE: OR 1.08 (95% CI 0.75–1.56)

Seeto et al. [31]	2023	• Romosozumab versus placebo:
		○ Three‐point MACE: OR 1.25 (95% CI 0.79–2.11)
		○ Four‐point MACE: OR 1.31 (95% CI 0.89–2.02)
		○ Five‐point MACE: 1.35 (95% CI 0.92–2.12)
		○ Myocardial infarction: OR 1.44 (95% CI 0.44–4.90)
		○ Stroke: OR 1.21 (95% CI 0.50–2.98)

Kobayashi et al. [32]	2024	• Romosozumab versus placebo:
		○ Three‐point MACE: RR 1.19 (95% CI 0.71–1.99)
		○ Four‐point MACE: RR 1.36 (95% CI 0.87–2.12)
		○ Five‐point MACE: RR 1.34 (95% CI 0.88–2.02)
		○ Myocardial infarction: RR 1.45 (95% CI 0.54–3.92)
		○ Cardiovascular death: RR 1.08 (95% CI 0.55–2.11)
		○ Stroke: RR 1.09 (95% CI 0.29–4.11)
		○ Heart failure: RR 1.71 (95% CI 0.74–3.98)
		○ Atrial fibrillation: RR 1.01 (95% CI 0.36–2.82)

Wong et al. [33]	2024	• Romosozumab compared to active treatment or placebo
		○ Within 12 months of treatment:
		▪ Serious cardiovascular event: OR 1.21 (95% CI 0.90–1.63)
		▪ Cardiovascular death: OR 1.24 (95% CI 0.76–2.04)
		▪ Overall: OR 1.22 (95% CI 0.95–1.57)
		○ Within 12 months of treatment (and 24 months of antiresorptive therapy in Asian population):
		▪ Serious cardiovascular event: OR 1.09 (95% CI 0.40–2.96)

Cheng et al. [34]	2025	• Romosozumab versus placebo:
		○ Cardiovascular mortality: RR 1.08 (95% CI 0.57–2.04)
		○ Cardiovascular event: RR 1.10 (0.73–1.65)

Ferrer et al. [35]	2025	• Romosozumab versus alternative treatment:
		○ Cardiovascular adverse events: OR 1.24 (95% CI 0.89–1.71)

Considering the available data from the randomized controlled trials, the absolute increase between romosozumab and control groups for cardiovascular events is estimated at 0.4%, translating to a number needed to harm of 250 [19]. Comparatively, however, the number needed to treat for vertebral, nonvertebral, or hip fractures from the ARCH trial, are 18, 53, and 84, respectively [21]. Quite reassuringly, data from the FRAME and ARCH trials noted a reduction in fracture rates exceeding the risk for adverse cardiovascular events at all points in time (and further widening over time) [19]. Cheng et al. report the pooled cardiovascular mortality rate from romosozumab to be 548 per 100,000, in keeping with the global range for mortality from cardiovascular disease for females between the ages of 50–64 following a 31‐year trend [34].

### 4.3. Biological Plausibility

Presently, there is no convincing evidence for establishing a biological basis for cardiovascular disease with sclerostin inhibition. Notably, Phase I–III trials did not demonstrate significant changes in heart rate, blood pressure, biochemical markers, or electrocardiographic variables [19]. Moreover, additional antisclerostin monoclonals, such as blosozumab and setrusumab (the latter under investigation for osteogenesis imperfecta), have not demonstrated an increase for cardiovascular events [19]. AMGEN (company filing for romosozumab) extensively investigated romosozumab in animal models, attempting to identify a mechanism of rapid cardiovascular events [20]. At ten‐fold the serum level in postmenopausal women, the company failed to induce vasoconstriction [21]. With such ambiguity, it should not come as a surprise that during a 2023 American Society for Bone and Mineral Research debate, merely 30% of members indicated a belief that a causal relationship exists between sclerostin inhibition and cardiovascular risk [12].

While sclerosteosis has undetectable serum sclerostin levels, it is notable that an increase in cardiovascular events has not been depicted; this is limited, however, by the shortened median survival time (from noncardiac death) [21]. While patients with van Buchem disease have markedly reduced serum sclerostin, these individuals often have a near‐normal life expectancy and also do not appear to have an excess of cardiovascular events [21]. The rarity of these two conditions (each with no more than a couple hundred cases documented globally) restricts the generalizability and precludes definitive conclusions regarding vascular effects of sclerostin suppression. Romosozumab, however, binds to serum sclerostin (rather than suppressing production); hence, levels are often unchanged. Despite numerous studies within the medical literature, the correlation between serum sclerostin and cardiovascular health remains inconclusive. Meta‐analyses have reported an absent, positive, or inverse correlation [20]. Certain authors have suggested serum sclerostin to be a surrogate marker for cardiovascular health, correlating with coronary artery calcification, insulin resistance, hyperlipidemia, and arterial stiffness [35]. On the contrary, emerging evidence suggests that sclerostin levels may increase as a compensatory mechanism [36]. As an example, in patients with renal disease and carotid artery atherosclerosis, serum sclerostin levels are often elevated; as sclerostin is expressed in numerous tissues, this does not appear to be due to reduced renal clearance, but rather, upregulation [37, 38]. Hypersclerostinemia has also been noted in patients with Type 2 diabetes mellitus and increased carotid intima–media thickness, hypothesized to prevent the progression of atherosclerosis [36].

Sclerostin is expressed in various tissues, including heart, cartilage, kidney, and vascular smooth muscle [21]. It has furthermore been identified in vascular and valvular calcification, generating the hypothesis that it prevents plaque formation; animal studies, however, have failed to support this, and sclerostin has not been identified in fibrous caps of atherosclerotic plaque [19, 21]. Romosozumab exposure (more than 90‐fold exposure from area under the curve) to monkeys and rats has failed to induce vascular mineralization or morphological changes in calcification [20]. Similarly, ApoE −/− knockout mice do not appear to have altered morphologies (or incidences) of atherosclerotic plaque with sclerostin antibody administration [20]. Sclerostin expression throughout calcified vessels and valves has additionally been theorized to occur from an epiphenomenon of an ossification process; this is supported by the notion that other osteocyte markers may be expressed during vascular smooth muscle cell calcification [20]. Certain authors also note that sclerostin expression is predominantly expressed within the media (rather than intima) of the vasculature; this is important to consider, as at this location, adverse cardiovascular events would be slow to develop (rather than shortly after treatment initiation, as noted in the ARCH trial) [19].

Mimicking sclerosteosis, mouse models with *SOST* gene deletion have failed to induce vascular calcification [39]. With Mendelian randomization analyses, Bovjin et al. demonstrate *SOST* gene variants (rs7209826 and rs188810925) correlated with decreased arterial sclerostin expression, alongside significantly increased odds for cardiovascular events [27]. Contrarily, Holdsworth et al. analyzed these two variants, failing to find such an association after multivariate analyses; moreover, the authors investigated five further variants (rs9899889, rs1107748 and rs66838809, rs2741856, and rs7217502), once more failing to identify a correlation [40]. Among five gene variants studied by Zheng et al. (rs66838809, rs4793023, rs1107747, rs67449013, and rs80107551), the authors noted a statistically significant increased risk for cardiovascular events; Alcalde‐Herraiz et al. concur the correlation of cardiovascular events with rs66838809 and rs7220711 [41, 42].

### 4.4. Pharmacovigilance

While the FDA issued a warning regarding romosozumab usage in those with a myocardial infarction or cerebrovascular accident within the preceding year, the EMA lists these events as contraindications (irrespective of timing) [43]. Upon first approval in Japan, no warnings were listed; however, postmarketing concerns have led to a succession of events (from warnings to formal contraindications); moreover, the PMDA also requires a formal risk management plan and recommends monitoring of cardiovascular risks in such patients [32]. As a result, variations with regulatory recommendations and patterns of prescribing have led to heterogeneous patient populations with varying baseline cardiovascular risk, posing a challenge for direct comparison of outcomes across regions [20].

A pharmacovigilance study by Vestergaard Kvist et al. assessed the safety of romosozumab from extraction of the FDA Adverse Event Reporting System (FAERS) between January 2019 and December 2020 [42]. The authors noted a disproportionality signal with respect to myocardial infarction, stroke, and cardiovascular death from romosozumab. Of note, however, *n* = 1188 (59.5%) cases (of *n* = 1995) were from Japan, as were *n* = 164 (13.8%) (of *n* = 206) suspected MACE reports [12, 42]. The reported odds ratio of MACE was elevated in general (ROR 4.07, 95% CI 2.39–6.93), with ROR of MACE in Japan greater than the United States (Japan: ROR 3.65, 95% 1.98–6.38; the United States: ROR 1.83, 95% CI 0.84–4.00) [12, 42]. Importantly, however, the disproportionality signal in Japan could be a result of older patients and greater inclusion of male patients (who are at greater risk for cardiovascular disease) [12]. Moreover, as suggested by Kawaguchi et al., romosozumab was likely to have been used in higher‐risk patients in Japan for six months until a package insert was added in September 2019, leading to a relative selection bias among cases across the United States [12]. It should be noted, however, that there does not appear to be a clear reduction in MACE trends in Japan following the addition of the package insert [12].

An additional pharmacovigilance study by Chen et al. from FAERS during the period of January 2019 through December 2020 analyzed *n* = 1948 romosozumab‐related adverse events [44]. The authors, however, solely report cardiac failure (ROR 12.62, 95% CI 9.85–16.17) rather than MACE [44]. Another pharmacovigilance study from the Japanese Adverse Drug Event Report (JADER) database was reported by Kotake et al. [45]. The authors assessed the cardiovascular safety of *n* = 859 patients treated with romosozumab, noting a disproportionality signal for cardiac (ROR 5.6, 95% CI 4.5–6.9, *p* < 0.01) and cerebrovascular events (ROR 6.1, 95% CI 5.0–7.3, *p* < 0.01) (which was more frequent than other osteoporotic agents such as bisphosphonates, denosumab, or teriparatide), with *n* = 102 ischemic heart and *n* = 133 cerebrovascular events [45].

Evidently, however, pharmacovigilance studies are prone to bias from unmeasured underlying diseases, with overreliance on spontaneous reporting, along with a heterogeneous report of adverse events without a proper control group [14]. Furthermore, pharmacovigilance studies do not prove causality, nor do they provide an absolute number of overall treated patients [14]. A particular fallacy of pharmacovigilance studies is the notion that they evaluate coadministered cardioprotective drugs rather than comorbidities, due to a limitation of the FAERS database not containing comorbidity data [45].

### 4.5. Clinical Implications

The findings of this study should be considered hypothesis‐generating rather than practice‐changing. Although an association between romosozumab use and lower observed cardiovascular event rates was identified, the potential influence of immortal time bias, channeling bias, and residual confounding substantially limits causal interpretation. Accordingly, these results should not be used to modify current clinical practice or regulatory recommendations. Importantly, treatment decisions should balance cardiovascular safety concerns with the substantial fracture risk reduction associated with romosozumab. In patients at very high risk of fracture, the benefits of therapy may outweigh potential cardiovascular risks, particularly in the absence of recent cardiovascular events. From a regulatory perspective, our findings do not provide sufficient evidence to support removal or modification of the current black‐box warning but highlight the need for further evaluation.

### 4.6. Limitations

These findings must be interpreted in the context of potential biases inherent to observational data. The retrospective study design precludes causal inference. Despite the use of propensity score matching, residual and unmeasured confounding remains a significant limitation. Important variables relevant to cardiovascular risk (such as frailty, functional status, socioeconomic factors, detailed comorbidity burden, cardiovascular risk profiles, and physician prescribing behavior) were incompletely captured and may differ systematically between cohorts. As a result, these unmeasured factors may materially influence the observed associations, and the possibility that the findings are driven, at least in part, by residual confounding cannot be excluded.

Selection and channeling (prescribing) bias are important limitations. Given the known boxed cardiovascular warning associated with romosozumab, clinicians may preferentially prescribe this therapy to patients perceived to be at lower cardiovascular risk, while directing higher‐risk individuals toward alternative treatments. This results in systematic differences between treatment groups that may not be fully captured by measured covariates, even after propensity score matching. Consequently, this bias may have contributed to the observed protective associations by selecting a comparatively lower‐risk population for romosozumab exposure. The requirement for multiple administrations of romosozumab introduces a risk of immortal time bias, as patients must survive event‐free during this period to be classified as exposed. This exposure definition may have contributed substantially to the observed protective association, as it inherently selects for patients who remain event‐free long enough to meet inclusion criteria. Consequently, the magnitude of the observed reduction in cardiovascular events may be, at least in part, an artifact of study design rather than a true treatment effect. Ideally, this limitation would be addressed using a time‐dependent exposure model; however, such analyses were not feasible within the constraints of the TriNetX platform. As a result, our exposure definition represents a pragmatic approximation of treatment adherence but introduces bias that would be expected to favor a protective association. The magnitude of this bias is difficult to quantify but may partially account for the observed reduction in cardiovascular events. Although propensity score matching substantially improved covariate balance, complete balance was not achieved. Outcome definitions relied on selected ICD‐10 codes chosen to prioritize specificity for acute cardiovascular events. While this approach reduces misclassification from nonspecific coding, it may result in underascertainment and limit capture of the full clinical spectrum of disease. Accurate coding may also vary across institutions within the TriNetX network. Such misclassification is likely to tend to bias results toward the null, potentially underestimating true associations. Missing data were present for key variables, particularly laboratory measures such as LDL cholesterol and hemoglobin A1c, which were only available for a subset of patients. Analyses were conducted using available‐case data without imputation, and patients were retained despite incomplete data for certain variables. This approach may reduce the effectiveness of confounder adjustment and introduce residual confounding if missingness is not random. The study also lacked granular clinical data, including baseline bone mineral density, osteoporosis severity, fracture burden, functional status, mobility, physical activity, bone mineral density changes, fracture outcomes, medication adherence beyond prescription records, and detailed measures of cardiovascular disease severity. Consequently, differences in disease severity or mobility between cohorts may have influenced cardiovascular outcomes and could not be fully accounted for in the present analysis. We did not perform fracture‐specific subgroup analyses (such as hip fracture), and therefore, cannot exclude the possibility that differences in fracture severity, frailty, or associated mortality risk influenced the observed associations. Competing risks were not formally accounted for in this analysis. In particular, mortality represents a competing event for nonfatal cardiovascular outcomes, as patients who die are no longer at risk of experiencing subsequent events such as myocardial infarction, heart failure, or cerebrovascular accident. The use of Kaplan–Meier and Cox proportional hazards methods may therefore overestimate the incidence of nonfatal outcomes by treating competing events as noninformative censoring. As a result, the reported hazard estimates should be interpreted with caution, as they may be influenced by the absence of competing risk modeling. Finally, the TriNetX network is predominantly composed of U.S. healthcare data, which may limit generalizability to other populations and healthcare systems. Differences in patient demographics, healthcare and insurance access, coding practices, and prescribing patterns may influence the applicability of these findings to non‐U.S. settings.

## 5. Conclusion

In this real‐world propensity score–matched analysis, romosozumab exposure was associated with lower observed rates of MACEs compared with teriparatide/abaloparatide. However, these findings should be interpreted as associative rather than causal due to the observational design and potential for residual confounding, selection bias, and immortal time bias. While no clear increased cardiovascular risk signal was identified, these results are not sufficient to alter current regulatory recommendations. Further prospective and mechanistic studies are required to better define the cardiovascular safety profile of sclerostin inhibition.

## Author Contributions

Maxim John Levy Barnett led the study design, analysis, and drafting of the manuscript. Justin Lam assisted with data analysis, supervision, and revisions. Catherine Anastasopoulou supervised the project and approved the final version.

## Funding

No funding was received for this research.

## Disclosure

Permission was obtained to reuse Figure 1.

All authors have reviewed and approved the manuscript.

## Conflicts of Interest

The authors declare no conflicts of interest.

## Supporting Information

Additional supporting information can be found online in the Supporting Information section.

## Supporting information


**Supporting Information 1** Supporting Appendix 1 demonstrates the completed STROBE reporting checklist. The checklist documents where each recommended reporting item for the observational cohort study is addressed (including study design, data source, participant selection, exposure, outcome definitions, propensity score matching approach, handling of missing data, statistical methods, subgroup and sensitivity analyses, limitations interpretation, generalizability, and funding information). Supporting Appendix 2 demonstrates the baseline demographic and clinical characteristics of Cohort A and Cohort B before and after propensity score matching. Before matching, significant differences were observed in age, sex distribution, racial composition, cardiovascular comorbidities, and laboratory values, as reflected by *p* values and standardized differences; after matching, these imbalances were markedly reduced, with standardized differences approaching zero across variables, indicating successful cohort balance. Supporting Appendix 3 demonstrates the subgroup analyses at one‐, two‐, and five‐year follow‐up, showing that romosozumab was associated with consistent and statistically significant reductions in four‐point MACE, three‐point MACE, heart failure, death, and acute myocardial infarction (although log‐rank testing suggested significance for cerebrovascular accidents at one and two years, this was not supported by Cox regression and was not observed at five years, likely reflecting a low event rate). Supporting Appendix 4 demonstrates that, within the male subgroup, romosozumab was associated with numerically lower event rates for four‐point MACE, three‐point MACE, and hear failure; however, these differences did not reach statistical significance. Analyses for death, myocardial infarction, and cerebrovascular accidents were not performed due to an insufficient number of events. Supporting Appendix 5 demonstrates that, among female users, romosozumab was associated with consistent and statistically significant reductions across all major cardiovascular outcomes at one year. Both log‐rank and Cox regression analyses showed significant reductions in four‐ and three‐point MACE, heart failure, death, and myocardial infarction, and cerebrovascular accidents were also significantly lower despite the small number of events. Supporting Appendix 6 demonstrates that, in the 50–64‐year subgroup, romosozumab was associated with a statistically significant reduction in four‐point MACE based on both log‐rank and Cox regression analyses. Three‐point MACE and heart failure showed significance by log‐rank testing but were not confirmed by Cox regression, likely reflecting limited event counts, and insufficient events precluded analyses for death, acute myocardial infarction, and cerebrovascular accidents. Supporting Appendix 7 demonstrates that, in the subgroup aged > 65 years, romosozumab was associated with statistically significant reductions in four‐point MACE, three‐point MACE, heart failure, death, and acute myocardial infarction based on both log‐rank and Cox regression analyses. Cerebrovascular accidents showed a numerically lower hazard in the romosozumab cohort, although this did not reach statistical significance.

**Supporting Information 2** STROBE statement.

## Data Availability

Data are available on request from the authors.
