# Supplementary material for: Cardiovascular Outcomes Following Therapeutic Sclerostin Inhibition Compared With Alternative Anabolic Therapies: A Real‐World Propensity Score–Matched Analysis
Source: J Osteoporos. 2026 Jul 30;2026:2813928. doi: 10.1155/joos/2813928 (PMC13422644; doi:10.1155/joos/2813928)
Supplement: Supplementary file 1 — Supporting Information 1 Supporting Appendix 1 demonstrates the completed STROBE reporting checklist. The checklist documents where each recommended reporting item for the observational cohort study is addressed (including study design, data source, participant selection, exposure, outcome definitions, propensity score matching approach, handling of missing data, statistical methods, subgroup and sensitivity analyses, limitations interpretation, generalizability, and funding information). Supporting Appendix 2 demonstrates the baseline demographic and clinical characteristics of Cohort A and Cohort B before and after propensity score matching. Before matching, significant differences were observed in age, sex distribution, racial composition, cardiovascular comorbidities, and laboratory values, as reflected by p values and standardized differences; after matching, these imbalances were markedly reduced, with standardized differences approaching zero across variables, indicating successful cohort balance. Supporting Appendix 3 demonstrates the subgroup analyses at one‐, two‐, and five‐year follow‐up, showing that romosozumab was associated with consistent and statistically significant reductions in four‐point MACE, three‐point MACE, heart failure, death, and acute myocardial infarction (although log‐rank testing suggested significance for cerebrovascular accidents at one and two years, this was not supported by Cox regression and was not observed at five years, likely reflecting a low event rate). Supporting Appendix 4 demonstrates that, within the male subgroup, romosozumab was associated with numerically lower event rates for four‐point MACE, three‐point MACE, and hear failure; however, these differences did not reach statistical significance. Analyses for death, myocardial infarction, and cerebrovascular accidents were not performed due to an insufficient number of events. Supporting Appendix 5 demonstrates that, among female users, romosozumab was associated with [file JOOS-2026-2813928-s002.docx]

**Supplementary Appendix 1: STROBE STATEMENT**

STROBE Statement—Checklist of items that should be included in reports of ***cohort studies***

|  | Item No | Recommendation | Page No |
| --- | --- | --- | --- |
| **Title and abstract** | 1 | (*a*) Indicate the study’s design with a commonly used term in the title or the abstract | 1 |
|  |  | (*b*) Provide in the abstract an informative and balanced summary of what was done and what was found | 2 |
| Introduction | | | |
| Background/rationale | 2 | Explain the scientific background and rationale for the investigation being reported | 3-4 |
| Objectives | 3 | State specific objectives, including any prespecified hypotheses | 4 |
| Methods | | | |
| Study design | 4 | Present key elements of study design early in the paper | 4-7 |
| Setting | 5 | Describe the setting, locations, and relevant dates, including periods of recruitment, exposure, follow-up, and data collection | 5-6 |
| Participants | 6 | (*a*) Give the eligibility criteria, and the sources and methods of selection of participants. Describe methods of follow-up | 5-6 |
|  |  | (*b*) For matched studies, give matching criteria and number of exposed and unexposed | 5-7 |
| Variables | 7 | Clearly define all outcomes, exposures, predictors, potential confounders, and effect modifiers. Give diagnostic criteria, if applicable | 7 |
| Data sources/ measurement | 8* | For each variable of interest, give sources of data and details of methods of assessment (measurement). Describe comparability of assessment methods if there is more than one group | 7-8 |
| Bias | 9 | Describe any efforts to address potential sources of bias | 7 |
| Study size | 10 | Explain how the study size was arrived at | 7 |
| Quantitative variables | 11 | Explain how quantitative variables were handled in the analyses. If applicable, describe which groupings were chosen and why | 5-7 |
| Statistical methods | 12 | (*a*) Describe all statistical methods, including those used to control for confounding | 4-6, 8, 9 |
|  |  | (*b*) Describe any methods used to examine subgroups and interactions | 9-11 |
|  |  | (*c*) Explain how missing data were addressed | 6 |
|  |  | (*d*) If applicable, explain how loss to follow-up was addressed | N/A |
|  |  | (*e*) Describe any sensitivity analyses | 9-11 |
| Results | | |  |
| Participants | 13* | (a) Report numbers of individuals at each stage of study—eg numbers potentially eligible, examined for eligibility, confirmed eligible, included in the study, completing follow-up, and analysed | 7 |
|  |  | (b) Give reasons for non-participation at each stage | N/A |
|  |  | (c) Consider use of a flow diagram | N/A |
| Descriptive data | 14* | (a) Give characteristics of study participants (eg demographic, clinical, social) and information on exposures and potential confounders | 7 |
|  |  | (b) Indicate number of participants with missing data for each variable of interest | N/A |
|  |  | (c) Summarise follow-up time (eg, average and total amount) | 8-9 |
| Outcome data | 15* | Report numbers of outcome events or summary measures over time | 8-11 |

| Main results | 16 | (*a*) Give unadjusted estimates and, if applicable, confounder-adjusted estimates and their precision (eg, 95% confidence interval). Make clear which confounders were adjusted for and why they were included | 8-11 |
| --- | --- | --- | --- |
|  |  | (*b*) Report category boundaries when continuous variables were categorized | 8-11 |
|  |  | (*c*) If relevant, consider translating estimates of relative risk into absolute risk for a meaningful time period | 8-11 |
| Other analyses | 17 | Report other analyses done—eg analyses of subgroups and interactions, and sensitivity analyses | 8-11 |
| Discussion | | | |
| Key results | 18 | Summarise key results with reference to study objectives | 11 |
| Limitations | 19 | Discuss limitations of the study, taking into account sources of potential bias or imprecision. Discuss both direction and magnitude of any potential bias | 19,20 |
| Interpretation | 20 | Give a cautious overall interpretation of results considering objectives, limitations, multiplicity of analyses, results from similar studies, and other relevant evidence | 11-19 |
| Generalisability | 21 | Discuss the generalisability (external validity) of the study results | 19 |
| Other information | | | |
| Funding | 22 | Give the source of funding and the role of the funders for the present study and, if applicable, for the original study on which the present article is based | 1 |

*Give information separately for exposed and unexposed groups.
