# Supplementary material for: Cardiovascular Outcomes Following Therapeutic Sclerostin Inhibition Compared With Alternative Anabolic Therapies: A Real‐World Propensity Score–Matched Analysis
Source: J Osteoporos. 2026 Jul 30;2026:2813928. doi: 10.1155/joos/2813928 (PMC13422644; doi:10.1155/joos/2813928)
Supplement: Supplementary file 2 — Supporting Information 2 STROBE statement. [file JOOS-2026-2813928-s001.docx]

**Supplementary Appendix 2: Before and After Matching Characteristics of Cohorts A and B**

Supplementary Table 1: Baseline demographic and clinical characteristics of Cohort A and Cohort B before and after propensity score matching.

Before matching, significant differences were observed in age, sex distribution, racial composition, cardiovascular comorbidities, and laboratory values, as reflected by p-values and standardized differences; after matching, these imbalances were markedly reduced, with standardized differences approaching zero across variables, indicating successful cohort balance.

| **Variables** | **Before Matching** | | | | | | | | **After Matching** | | | | | | | |
| --- | --- | --- | --- | --- | --- | --- | --- | --- | --- | --- | --- | --- | --- | --- | --- | --- |
|  | Cohort A | Cohort B | Patients (n)  (Cohort A) | Patients (n)  (Cohort B) | % of Cohort  (Cohort A) | % of Cohort (Cohort B) | p-value | Std. diff. | Cohort A | Cohort B | Patients (n)  (Cohort A) | Patients (n) (Cohort B) | % of Cohort  (Cohort A) | % of Cohort  (Cohort B) | p-value | Std diff. |
| Age at Index (Years, Mean + SD) | 71.1 + 8.55 | 69.6 + 9.49 | 13,400 | 40,238 | 100% | 100% | < 0.0001 | 0.1753 | 71.1 + 8.55 | 71.2 + 8.6 | 13,400 | 13,400 | 100% | 100% | 0.5695 | 0.0069 |
| Female | - | - | 13,056 | 34,899 | 97.433% | 86.731% | < 0.0001 | 0.4043 | - | - | 13,056 | 13,051 | 97.433% | 97.396% | 0.874 | 0.0024 |
| Not Hispanic or Latino | - | - | 10,477 | 31,378 | 77.963% | 77.981% | 0.9646 | 0.0004 | - | - | 10,447 | 10,544 | 77.963% | 78.687% | 0.1504 | 0.0176 |
| White | - | - | 10,154 | 30,721 | 75.776% | 76.348% | 0.1780 | 0.0134 | - | - | 10,154 | 10,189 | 75.776% | 76.037% | 0.6171 | 0.0061 |
| Unknown Ethnicity | - | - | 2,500 | 7,104 | 18.657% | 17.655% | 0.0088 | 0.0260 | - | - | 2,500 | 2,448 | 18.657% | 18.269% | 0.4130 | 0.0100 |
| Asian | - | - | 1,357 | 3,185 | 10.127% | 7.915% | < 0.0001 | 0.0773 | - | - | 1,357 | 1,342 | 10.127% | 10.015% | 0.7608 | 0.0037 |
| Male | - | - | 343 | 5,326 | 2.56% | 13.236% | < 0.0001 | 0.4038 | - | - | 343 | 346 | 2.56% | 2.582% | < 0.9078 | 0.0014 |
| Black or African American | - | - | 184 | 957 | 1.373% | 2.378% | < 0.0001 | 0.0741 | - | - | 184 | 142 | 1.373% | 1.06% | 0.0193 | 0.0286 |
| Diseases of the circulatory system | - | - | 9,004 | 27,609 | 67.194% | 68.614% | 0.0022 | 0.0304 | - | - | 9,004 | 8,971 | 67.194% | 66.948% | 0.6680 | 0.0052 |
| Essential Hypertension | - | - | 6,344 | 19,971 | 47.343% | 49.632% | < 0.0001 | 0.0458 | - | - | 6,344 | 6,366 | 47.343% | 47.507% | 0.7878 | 0.0033 |
| Chronic Kidney Disease | - | - | 1,563 | 4,917 | 11.664% | 12.22% | 0.0874 | 0.0171 | - | - | 1,563 | 1,458 | 11.664% | 10.881% | 0.0426 | 0.0248 |
| Family history of ischemic heart disease and other diseases of the circulatory system | - | - | 1,304 | 3,049 | 9.731% | 7.577% | < 0.0001 | 0.0767 | - | - | 1,304 | 1,197 | 9.731% | 8.933% | 0.0246 | 0.0275 |
| Tobacco use | - | - | 467 | 1,705 | 3.485% | 4.237% | 0.0001 | 0.0390 | - | - | 467 | 397 | 3.485% | 2.963% | 0.0155 | 0.0296 |
| Family history of stroke | - | - | 294 | 660 | 2.194% | 1.64% | < 0.0001 | 0.0404 | - | - | 294 | 231 | 2.194% | 1.724% | 0.0055 | 0.0339 |
| LDL-Cholesterol (Mean +/- SD mg/dL) | 102 + 34.1 | 98.5+ 36 | 8,264 | 21,251 | 61.672% | 52.813% | < 0.0001 | 0.1024 | 102 + 34.1 | 100 + 35 | 8,264 | 8,251 | 61.672% | 61.575% | < 0.0001 | 0.0608 |
| HbA1c (mean +/- SD %) | 5.71 + 0.77 | 5.81 + 0.969 | 6,408 | 17,482 | 47.821% | 43.446% | < 0.0001 | 0.1202 | 5.71 + 0.777 | 5.79 + 0.905 | 6,408 | 6,363 | 47.821% | 47.485% | < 0.0001 | 0.1017 |
| Cardiac Medications | - | - | 10,525 | 32,113 | 78.545% | 79.808% | 0.0017 | 0.0311 | - | - | 10,525 | 10,560 | 78.545% | 78.806% | 0.6017 | 0.0064 |

**Supplementary Appendix 3: Subgroup Analysis at 1, 2 and 5 Years**

- Four-Point MACE:
  - Year 1:
    - Number of events: Cohort A: n = 222; Cohort B: n = 466
    - Survival Probability: Cohort A: n = 98.1%; Cohort B: n = 95.937%
    - Log-Rank Test: χ² = 106.658, p < 0.0001
    - Hazard Ratio: 0.441 (95% CI 0.376-0.517, p < 0.0001)
  - Year 2:
    - Number of events: Cohort A: n = 397; Cohort B: n = 737
    - Survival Probability: Cohort A: n = 95.7%; Cohort B: n = 92.7%
    - Log-Rank Test: χ² = 106.15, p < 0.0001
    - Hazard Ratio:0.532 (95% CI 0.471-0.601, p < 0.0001)
  - Year 5:
    - Number of events: Cohort A: n = 594; Cohort B: n = 1,147
    - Survival Probability: Cohort A: 86.118%; Cohort B: 83.411%
    - Log-Rank Test: χ² = 80.545, p < 0.0001
    - Hazard Ratio: 0.634 (95% CI 0.573-0.701, p < 0.0001)
- Three-Point MACE
  - Year 1:
    - Number of events: Cohort A: n = 686; Cohort B: n = 2,008
    - Survival Probability: Cohort A: 94.5%; Cohort B: 91.528%
    - Log-Rank Test: χ² = 92.27, p < 0.0001
    - Hazard Ratio: 0.624 (95% CI 0.567-0.688, p < 0.0001)
  - Year 2:
    - Number of events: Cohort A: n = 870; Cohort B: n = 1,254
    - Survival Probability: Cohort A: 91.953%; Cohort B: 88.518%
    - Log-Rank Test: χ² = 91.504, p < 0.0001
    - Hazard Ratio: 0.658 (95% CI 0.603-0.717, p < 0.0001)
  - Year 5:
    - Number of events: Cohort A: n = 1,045; Cohort B: n = 1,628
    - Survival Probability: Cohort A: 83.568%; Cohort B: 80.173%
    - Log-Rank Test: χ² = 88.538, p < 0.0001
    - Hazard Ratio: 0.688 (95% CI 0.636-0.744, p < 0.0001)
- Heart Failure:
  - Year 1:
    - Number of events: Cohort A: n = 618; Cohort B: n = 887
    - Survival Probability: Cohort A: 95.057%; Cohort B: 92.545%
    - Log-Rank Test: χ² = 73.223, p < 0.0001
    - Hazard Ratio: 0.641 (95% CI 0.578-0.71, p = 0.0002)
  - Year 2:
    - Number of events: Cohort A: n = 773; Cohort B: n = 1,070
    - Survival Probability: Cohort A: 92.913%; Cohort B: 90.301%
    - Log-Rank Test: χ² = 64.849, p < 0.0001
    - Hazard Ratio: 0.685 (95% CI 0.625-0.752, p < 0.0001)
  - Year 5:
    - Number of events: Cohort A: n = 927; Cohort B: n = 1,420
    - Survival Probability: Cohort A: 85.467%; Cohort B: 82.739%
    - Log-Rank Test: χ² = 70.224, p < 0.0001
    - Hazard Ratio: 0.701 (95% CI 0.645-0.762, p < 0.0001)
- Death:
  - Year 1:
    - Number of events: Cohort A: n = 104; Cohort B: n = 246
    - Survival Probability: Cohort A: 99.078%; Cohort B: 97.803%
    - Log-Rank Test: χ² = 68.265, p < 0.0001
    - Hazard Ratio: 0.394 (95% CI 0.313-0.495, p < 0.0001)
  - Year 2:
    - Number of events: Cohort A: n = 233; Cohort B: n = 426
    - Survival Probability: Cohort A: 97.32%; Cohort B: 95.65%
    - Log-Rank Test: χ² = 55.007, p < 0.0001
    - Hazard Ratio: 0.551 (95% CI 0.47-0.646, p < 0.0001)
  - Year 5:
    - Number of events: Cohort A: n = 385; Cohort B: n = 777
    - Survival Probability: Cohort A: 89.769%; Cohort B: 87.737%
    - Log-Rank Test: χ² = 46.997, p < 0.0001
    - Hazard Ratio: 0.65 (95% CI 0.574-0.736, p < 0.0001)
- Acute Myocardial Infarction:
  - Year 1:
    - Number of events: Cohort A: n = 103 ; Cohort B: n = 197
    - Survival Probability: Cohort A: 99.14%; Cohort B: 98.307%
    - Log-Rank Test: χ² = 37.094, p < 0.0001
    - Hazard Ratio: 0.484 (95% CI 0.382-0.615, p = 0.0025)
  - Year 2:
    - Number of events: Cohort A: n = 161; Cohort B: n = 305
    - Survival Probability: Cohort A: 98.334%; Cohort B: 96.974%
    - Log-Rank Test: χ² = 46.789, p < 0.0001
    - Hazard Ratio: 0.519 (95% CI 0.429-0.629, p = 0.0271)
  - Year 5:
    - Number of events: Cohort A: n = 219; Cohort B: n = 429
    - Survival Probability: Cohort A: 95.576%; Cohort B: 93.804%
    - Log-Rank Test: χ² = 37.066, p < 0.0001
    - Hazard Ratio: 0.603 (95% CI 0.511-0.711, p = 0.0054)
- Cerebrovascular Accident:
  - Year 1:
    - Number of events: Cohort A: n = 16; Cohort B: n = 31
    - Survival Probability: Cohort A: 99.867%; Cohort B: 99.741%
    - Log-Rank Test: χ² = 5.801, p = 0.0160
    - Hazard Ratio: 0.484 (95% CI 0.265-0.885, p = 0.1007)
  - Year 2:
    - Number of events: Cohort A: n = 19; Cohort B: n = 40
    - Survival Probability: Cohort A: 99.828%; Cohort B: 99.627%
    - Log-Rank Test: χ² = 8.112, p = 0.0044
    - Hazard Ratio: 0.461 (95% CI 0.267-0.796, p = 0.7498)
  - Year 5:
    - Number of events: Cohort A: n = 23; Cohort B: n = 38
    - Survival Probability: Cohort A: 99.71%; Cohort B: 99.607%
    - Log-Rank Test: χ² = 3.173, p = 0.0749
    - Hazard Ratio: 0.626 (95% CI 0.372-1.053, p = 0.1048)

Supplementary Appendix 2: Subgroup Analyses at One, Two and Five Years.

Romosozumab was associated with consistent and statistically significant reductions in four-point MACE, three-point MACE, heart failure, death, and acute myocardial infarction (Although log-rank testing suggested significance for cerebrovascular accidents at one and two years, this was not supported by Cox regression and was not observed at five years, likely reflecting a low event rate).

**Supplementary Appendix 4: Subgroup Analysis Male Cohort**

- Number of participants prior to matching:
  - Cohort A: n =343; Cohort B: n = 5,328
- Number of participants after matching:
  - Cohort A: n = 339; Cohort B: n = 339
- Four-Point MACE:
  - Number of events: Cohort A: n = 12; Cohort B: n = 20
  - Survival Probability: Cohort A: 95.9%; Cohort B: 93.2%
  - Log-Rank Test: χ² = 3.08, p = 0.079
  - Hazard Ratio: 0.532 (95% CI 0.26-1.09, p = 0.0056)
- Three-Point MACE:
  - Number of events: Cohort A: n = 39; Cohort B: n = 37
  - Survival Probability: Cohort A: 87.1%; Cohort B: 87.6%
  - Log-Rank Test: χ² = 0.07, p = 0.80
  - Hazard Ratio: 0.943 (95% CI 0.60-1.48, p = 0.0031)
- Heart Failure:
  - Number of events: Cohort A: n = 38; Cohort B: n = 34
  - Survival Probability: Cohort A: 87.5%; Cohort B: 88.6%
  - Log-Rank Test: χ² = 0.00, p = 0.992
  - Hazard Ratio: 1.003 (95% CI 0.63-1.59, p = 0.0048)
- Death, Acute Myocardial Infarction and Cerebrovascular Accident:
  - Too few events to compute stable survival estimate or hazard ratio

Supplementary Appendix 3: Subgroup Analysis of Male Cohort.

Within the male subgroup, romosozumab was associated with numerically lower event rates for four-point MACE, three-point MACE, and hear failure; however, these differences did not reach statistical significance. Analyses for death, myocardial infarction, and cerebrovascular accidents were not performed due to an insufficient number of events.

**Supplementary Appendix 5: Subgroup Analysis Female Cohort**

- Number of participants prior to matching:
  - Cohort A: n = 13,055; Cohort B: n = 34,863
- Number of participants after matching:
  - Cohort A: n = 13,055; Cohort B: n = 3,055
- Four-Point MACE:
  - Number of events: Cohort A: n = 210; Cohort B: n = 243
  - Survival Probability: Cohort A: 98.16%; Cohort B: 95.96%
  - Log-Rank Test: χ² = 109.3 , p < 0.0001
  - Hazard Ratio: 0.428 (95% CI 0.36-0.51, p < 0.0001)
- Three-Point MACE:
  - Number of events: Cohort A: n = 645; Cohort B: n = 997
  - Survival Probability: Cohort A: 94.70%; Cohort B: 91.45%
  - Log-Rank Test: χ² = 109.8, p < 0.0001
  - Hazard Ratio: 0.592 (95% CI 0.54-0.65, p < 0.0001)
- Heart Failure:
  - Number of events: Cohort A: n = 578; Cohort B: n = 864
  - Survival Probability: Cohort A: 95.3%; Cohort B: 92.6%
  - Log-Rank Test: χ² = 83.4, p < 0.0001
  - Hazard Ratio: 0.615 (95% CI 0.55-0.68, p < 0.0001)
- Death:
  - Number of events: Cohort A: n = 578; Cohort B: n = 230
  - Survival Probability: Cohort A: 99.12%; Cohort B: 97.89%
  - Log-Rank Test: χ² = 64.0, p < 0.0001
  - Hazard Ratio: 0.393 (95% CI 0.531-0.50, p < 0.0001)
- Acute Myocardial Infarction
  - Number of events: Cohort A: n = 99; Cohort B: n = 194
  - Survival Probability: Cohort A: 99.15%; Cohort B: 98.29%
  - Log-Rank Test: χ² = 38.6, p < 0.0001
  - Hazard Ratio: 0.473 (95% CI 0.36-0.60, p = 0.0002)
- Cerebrovascular Accident:
  - Number of events: Cohort A: n = 15; Cohort B: n = 39
  - Survival Probability: Cohort A: 99.87%; Cohort B: 99.67%
  - Log-Rank Test: χ² = 12.3, p = 0.0004
  - Hazard Ratio: 0.36 (95% CI 0.20-0.65, p = 0.045)

Supplementary Appendix 4: Subgroup Analysis of Female Cohort.

Among female users, romosozumab was associated with consistent and statistically significant reductions across all major cardiovascular outcomes at one year. Both log-rank and Cox regression analyses showed significant reductions in four- and three-point MACE, heart failure, death, and myocardial infarction, and cerebrovascular accidents were also significantly lower despite the small number of events.

**Supplementary Appendix 6: Subgroup Analysis Ages 50-64**

- Number of participants prior to matching:
  - Cohort A: n = 2,022; Cohort B: n = 8,138
- Number of participants after matching:
  - Cohort A: n = 2,020; Cohort B: n = 2,020
- Four-Point MACE:
  - Number of events: Cohort A: n = 12; Cohort B: n = 35
  - Survival Probability: Cohort A: 99.3%; Cohort B: 97.95%
  - Log-Rank Test: χ² = 4.782 , p = 0.0002
  - Hazard Ratio: 0.308 (95% CI 0.16-0.594, p = 0.0288)
- Three-Point MACE:
  - Number of events: Cohort A: n = 36; Cohort B: n = 69
  - Survival Probability: Cohort A: 98.1%; Cohort B: 96.0%
  - Log-Rank Test: χ² = 0.031 , p = 0.0002
  - Hazard Ratio: 0.474 (95% CI 0.316-0.031, p = 0.8605)
- Heart Failure:
  - Number of events: Cohort A: n = 33; Cohort B: n = 57
  - Survival Probability: Cohort A: 98.25%; Cohort B: 96.69%
  - Log-Rank Test: χ² = 0.051, p = 0.0029
  - Hazard Ratio: 0.527 (95% CI 0.343-0.809, p = 0.8218)
- Death, Acute Myocardial Infarction and Cerebrovascular Accident:
  - Too few events to compute stable survival estimate or hazard ratio

Supplementary Appendix 5: Subgroup Analysis Ages 50-64 Years.

In the 50–64-year subgroup, romosozumab was associated with a statistically significant reduction in four-point MACE based on both log-rank and Cox regression analyses. Three-point MACE and heart failure showed significance by log-rank testing but were not confirmed by Cox regression, likely reflecting limited event counts, and insufficient events precluded analyses for death, acute myocardial infarction, and cerebrovascular accidents.

**Supplementary Appendix 7: Subgroup Analysis Age > 65**

- Number of participants prior to matching:
  - Cohort A: n = 11,380; Cohort B: n = 32,110
- Number of participants after matching:
  - Cohort A: n = 11,379; Cohort B: n = 11,379
- Four-Point MACE:
  - Number of events: Cohort A: n = 210; Cohort B: n = 421
  - Survival Probability: Cohort A: 97.891%; Cohort B: 95.706%
  - Log-Rank Test: χ² = 87.752 , p < 0.0001
  - Hazard Ratio: 0.462 (95% CI 0.392-0.545, p < 0.0001)
- Three-Point MACE:
  - Number of events: Cohort A: n = 650; Cohort B: n = 925
  - Survival Probability: Cohort A: 93.871%; Cohort B: 90.917%
  - Log-Rank Test: χ² = 74.809 , p < 0.0001
  - Hazard Ratio: 0.645 (95% CI 0.583-0.713, p < 0.0001)
- Heart Failure:
  - Number of events: Cohort A: n = 585; Cohort B: n = 812
  - Survival Probability: Cohort A: 94.499%; Cohort B: 92.021%
  - Log-Rank Test: χ² = 58.17, p < 0.0001
  - Hazard Ratio: 0.663 (95% CI 0.596-0.738, p < 0.0001)
- Death:
  - Number of events: Cohort A: n = 99; Cohort B: n = 228
  - Survival Probability: Cohort A: 98.971%; Cohort B: 97.616%
  - Log-Rank Test: χ² = 60.364, p < 0.0001
  - Hazard Ratio: 0.405 (95% CI 0.32-0.513, p = 0.0001)
- Acute Myocardial Infarction
  - Number of events: Cohort A: n = 96; Cohort B: n = 187
  - Survival Probability: Cohort A: 99.059%; Cohort B: 98.122%
  - Log-Rank Test: χ² = 36.502, p < 0.0001
  - Hazard Ratio: 0.476 (95% CI 0.372-0.609, p = 0.0006)
- Cerebrovascular Accident:
  - Number of events: Cohort A: n = 16; Cohort B: n = 26
  - Survival Probability: Cohort A: 99.844%; Cohort B: 99.745%
  - Log-Rank Test: χ² = 3.093, p = 0.0786
  - Hazard Ratio: 0.576 (95% CI 0.309-1.074, p = 0.1003)

Supplementary Appendix 6: Subgroup Analysis Ages > 65 Years.

In the subgroup aged >65 years, romosozumab was associated with statistically significant reductions in four-point MACE, three-point MACE, heart failure, death, and acute myocardial infarction based on both log-rank and Cox regression analyses. Cerebrovascular accidents showed a numerically lower hazard in the romosozumab cohort, although this did not reach statistical significance.
